# Intramolecular interactions in the polar headgroup of sphingosine: serinol†
†Electronic supplementary information (ESI) available: *Ab initio* parameters for serinol conformers within 1000 cm^–1^, measured transition frequencies, typical *a*-type transition for conformer **aa1**, interconversion barriers and possible tunnelling pathways. See DOI: 10.1039/c5cc09423b



**DOI:** 10.1039/c5cc09423b

**Published:** 2016-01-04

**Authors:** Donatella Loru, Isabel Peña, José L. Alonso, M. Eugenia Sanz

**Affiliations:** a Department of Chemistry , King's College London , London , SE1 1DB , UK . Email: maria.sanz@kcl.ac.uk ; Tel: +44(0)2078487509; b Grupo de Espectroscopía Molecular (GEM) , Edificio Quifima , Laboratorios de Espectroscopia y Bioespectroscopia. Unidad Asociada CSIC , Parque Científico Uva , Universidad de Valladolid , Paseo de Belén 5 , 47011 Valladolid , Spain

## Abstract

The intramolecular interactions in the lipid sphingosine have been elucidated through the investigation of the amino alcohol serinol which mimics its polar headgroup. Intricate networks of intramolecular hydrogen bonds involving the hydroxyl groups and the amino group contribute to the stabilisation of five different conformations observed in the broadband rotational spectrum.

## Abstract 2

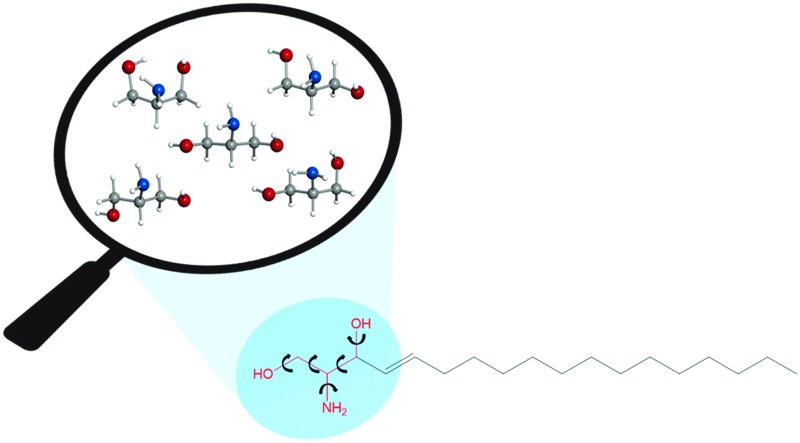
Intramolecular interactions in sphingosine have been elucidated through the investigation of its polar headgroup serinol.

Sphingosine is a lipid ubiquitous in eukaryotic cells, and it is also the building block of sphingolipids, which have been receiving increasing attention due to their participation in almost all processes related to cell regulation and signalling.^1^ In particular, sphingosine has a prominent role in cell death (apoptosis), differentiation and growth,^1–5^ and is involved in neurodegenerative diseases and cancer.^1,6–8^ Sphingosine is formed by an amino alcohol headgroup, with two hydroxyl groups and one amino group, attached to a long unsaturated hydrocarbon chain of typically 18 carbon atoms (see Scheme 1). The amino group can be protonated, but at the physiological pH it is mainly in the neutral form,^9,10^ which confers on sphingosine the ability to transfer freely among membranes and translocate, characteristics that are believed to be key for its biological function.

**Scheme 1:**
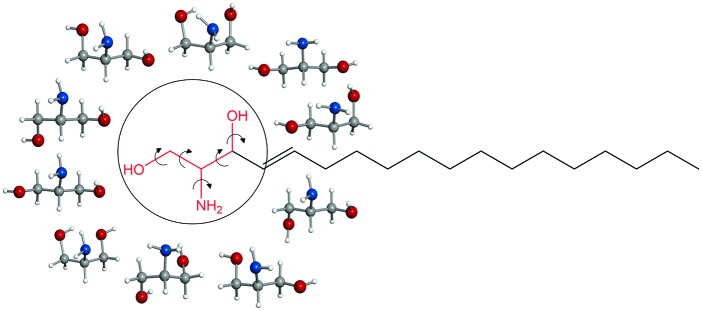


Intramolecular hydrogen bonds are postulated to be responsible for the existence of sphingosine as neutral at the physiological pH.^9,10^ The functional groups in the sphingosine head are expected to establish a variety of hydrogen bonds, as both –OH and –NH_2_ can act as proton donors and acceptors. Hydrogen bonds can also be established between the headgroups of neighbouring sphingosine molecules. Predominance of intermolecular hydrogen bonds has been proposed to lead to the formation of sphingosine aggregates, which are associated with sphingolipid storage disorders (one of the causes of neurodegenerative diseases).^10,11^ It has also been argued^11^ that strong hydrogen bonding in sphingolipid headgroups could be related to the formation of lipid rafts, to which proteins can attach and be transported in cells.^12^

A comprehensive understanding of the intra- and intermolecular interactions of sphingosine should start by elucidating relevant hydrogen bonds established within the polar headgroup, and this entails the identification of sphingosine conformations through a spectroscopic technique. Sphingosine is quite a large molecule (C_18_H_37_NO_2_) with many single bonds, and it is expected to present such a huge number of conformations that a detailed spectroscopic analysis would be an almost intractable problem. Not surprisingly there are no studies on the intramolecular interactions of sphingosine at an atomic level. By considering that the intramolecular interactions take place within the polar groups in the sphingosine head, with no involvement of the hydrocarbon tail, we have tackled the study of sphingosine hydrogen bonds through the investigation of its headgroup, the amino alcohol serinol. Even a relatively small molecule such as serinol is expected to exist in many conformations due to its torsional flexibility and ability to form different types of hydrogen bonds. Up to 135 conformers were found in a first exploration of the potential energy surface using the AM1 semi-empirical method. Subsequent optimisation^13^ using *ab initio* second order Moller perturbation theory (MP2) with the 6-311++G(d,p) basis set yielded 22 structures with energies below 1000 cm^–1^ (see Scheme 1, Table 1, and Table S1 in the ESI†). The experimental characterization of individual conformers of serinol requires a high resolution technique such as rotational spectroscopy. In this work chirped-pulse Fourier transform microwave (CP-FTMW) spectroscopy^14^ has been coupled with a laser ablation method that brings solid serinol intact into the gas phase to investigate its complex conformational behaviour. Details of the experimental setup are given elsewhere.^15,16^

**Table 1:** *Ab initio* ^*a*^ spectroscopic parameters for the predicted conformers of serinol with energies within 500 cm^–1^

MP2	**ga1**	**ga2**	**ga3**	**ga4**	**gG1**	**gG2**	**aa1**	**aa2**	**ag1**	**gg1**
*A* ^*b*^ (MHz)	6084.2	5996.5	6042.6	6033.1	4242.0	4094.0	7738.4	7642.0	5361.2	4172.4
*B* (MHz)	2278.9	2272.6	2258.1	2235.3	3134.0	3237.3	1977.6	1951.8	2387.1	3166.5
*C* (MHz)	1997.3	1977.5	1983.2	1951.3	2550.2	2479.7	1699.4	1686.4	1775.3	2195.6
*χ*_aa_ (MHz)	–0.34	–4.17	–0.68	–3.99	–2.60	–2.54	–3.53	–3.42	–4.22	1.86
*χ*_bb_ (MHz)	2.44	2.54	2.47	2.57	1.45	0.01	1.72	1.74	2.32	–0.20
*χ*_cc_ (MHz)	–2.10	1.64	–1.79	1.43	1.15	2.53	1.80	1.68	1.91	–1.66
*μ*_a_ (D)	–1.7	4.2	1.2	–2.0	–0.2	2.1	3.3	–1.9	3.3	1.9
*μ*_b_ (D)	0.3	–0.8	1.4	0.0	–2.6	–0.3	–1.0	–0.4	3.2	2.2
*μ*_c_ (D)	0.8	–1.4	3.0	0.9	–1.5	1.8	1.7	–0.1	1.2	0.5
Δ*E*_MP2+ZPC_ (cm^–1^)	0	215	248	443	146	503	298	494	584	478
Δ*G* ^298^ (cm^–1^)	0	159	276	420	115	499	231	457	495	450

^*a*^Optimised structures at the MP2/6-311++G(d,p) level, labelled according to the values of the ∠O_1_CCC angle (first label) and the ∠CCCO_2_ angle (second label) as **G** (+60°), **g** (–60°) and **a** (180°).

^*b*^ *A*, *B*, *C* are the rotational constants; *χ*_aa_, *χ*_bb_, and *χ*_cc_ are the ^14^N nuclear quadrupole coupling constants; *μ*_a_, *μ*_b_, *μ*_c_ are the electric dipole moment components; Δ*E* and Δ*G* are the MP2/6-311++G(d,p) electronic energies including the zero-point correction, and Gibbs free energies (298 K), respectively.

As anticipated from the large number of low-energy predicted conformers, the 6 to 18 GHz broadband rotational spectrum of serinol (Fig. 1) showed hundreds of lines. Careful analysis revealed rotational transitions belonging to five different rotamers, labelled I to V. All assigned transitions appeared to be split into several components (see the inset of Fig. 1), consistent with the nuclear quadrupole interaction expected from a molecule with a ^14^N nucleus. The measured transitions (see Tables S2–S6 in the ESI†) were fit^17^ to the semirigid rotor Hamiltonian,^18^ supplemented with a term to account for the quadrupole coupling interaction.^19^ In addition to the nuclear hyperfine structure, all a-type transitions of rotamer IV appeared as doublets separated by approximately 600 kHz (see Fig. S1 in the ESI†). No additional splittings were observed for any of the b- and c-type transitions of this rotamer. This suggests that rotamer IV tunnels between two equivalent configurations and that the *μ*_a_ dipole moment component inverts with the tunnelling motion (see the discussion below). Therefore to fit these transitions it was assumed that the a-type transitions connect two very close vibrational levels arbitrarily labelled 0+ and 0– while b- and c-type transitions are pure rotational transitions within each vibrational state. The determined rotational and quadrupole coupling constants of the rotamers are listed in Table 2.

**Fig. 1:**
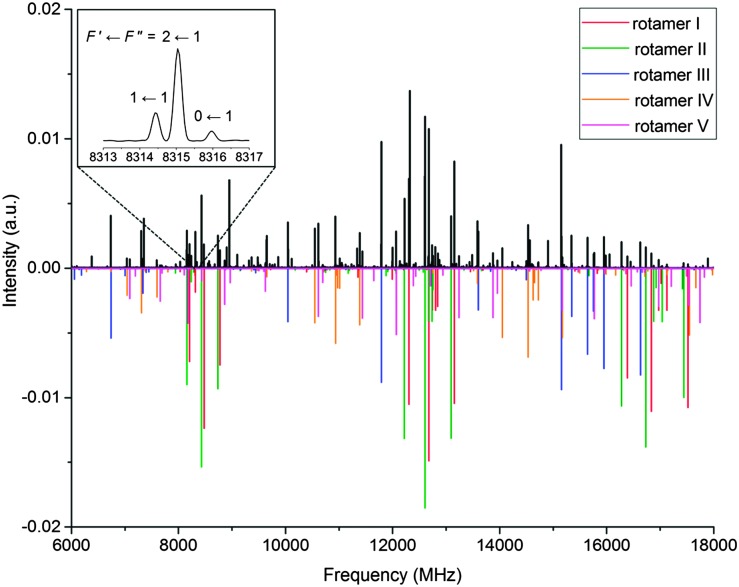
Broadband rotational spectrum of serinol in the 6–18 GHz frequency region. The upper trace shows the experimental spectrum, the lower trace is a simulation with the fitted rotational parameters and the experimental abundances. The inset shows the quadrupole coupling splitting of the 1_1,0_ ← 0_0,0_ transition of rotamer I.

**Table 2:** Experimental spectroscopic parameters determined for the observed conformers of serinol

	I	II	III	IV	V
*A* ^*a*^ (MHz)	6049.9322(41)^*e*^	5981.6740(48)	4208.5954(10)	7679.4716(36)^*f*^	5327.3056(33)
*B* (MHZ)	2265.0200(11)	2257.0848(12)	3130.7067(12)	1968.9155(10)	2367.0593(11)
*C* (MHz)	1981.1930(13)	1965.8411(14)	2527.3601(15)	1689.0099(12)	1762.9563(12)
*Δ*_J_ (kHz)	0.482(46)	0.409(45)	0.920(90)	0.293(29)	0.636(43)
*Δ*_JK_ (kHz)	—	—	—	2.051(25)	–0.971(22)
*Δ*_K_ (kHz)	7.69(10)	6.34(11)	—	—	—
*χ*_aa_ (MHz)	–0.299(11)	–3.9203(77)	–2.3658(73)	–3.2357(72)	–3.8976(90)
*χ*_bb_ (MHz)	2.338(13)	2.388(13)	1.228(10)	1.640(14)	2.252(13)
*χ*_cc_ (MHz)	–2.039(13)	1.533(13)	1.138(10)	1.596(14)	1.646(13)
Δ*E* (MHz)	—	—	—	0.2943(20)	—
*σ* ^*b*^ (kHz)	14	14	13	14	13
*N* ^*c*^	60	66	62	92	62
*a*/*b*/*c* ^*d*^	y/y/y	y/y/y	y/y/y	y/y/y	y/y/y

^*a*^ *A*, *B*, and *C* are the rotational constants; *Δ*_J_, *Δ*_JK_, and *Δ*_K_ are the quartic centrifugal distortion constants; *χ*_aa_, *χ*_bb_, and *χ*_cc_ are ^14^N nuclear quadrupole coupling constants; Δ*E* is the difference in energy between the two tunnelling states of serinol IV.

^*b*^rms deviation of the fit.

^*c*^Number of hyperfine transitions.

^*d*^Yes (y) or no (n) observation of a-, b-, and c-type transitions.

^*e*^Standard error in parentheses in the units of the last digit.

^*f*^The rotational constants obtained for each tunneling state were the same, within experimental error, when fitted separately.

Conformational assignment of the observed rotamers to specific conformers of serinol is achieved by comparing experimental and theoretical values of the spectroscopic constants (Tables 1, 2, and Table S1, ESI†). While rotational constants (*A*, *B*, *C*) are directly related to the molecular mass distribution of each conformer, ^14^N nuclear quadrupole coupling constants (*χ*_aa_, *χ*_bb_, *χ*_cc_) provide information on the local orientation of the amino group within the conformer. Both should be consistent with *ab initio* values, even if only one of these tools is acting as the discriminating element. The predicted values of electric dipole moment components (*μ*_a_, *μ*_b_, *μ*_c_), which are experimentally expressed in terms of the observed selection rules and intensity of rotational transitions, can also be used in the identification of conformers.

Rotamers I and III have rotational and quadrupole coupling constants compatible only with those predicted for conformers **ga1** and **gG1**, respectively (see the footnote of Table 1 for labelling). Those of rotamer II could initially match those of conformer **ga2** or **ga4**. However, the intense a-, b- and c-type observed spectra unambiguously identify this rotamer as conformer **ga2**, for which sizable *μ*_a_, *μ*_b_ and *μ*_c_ electric dipole moments are predicted (see Table 1). Similarly, the values of the rotational and quadrupole coupling constants of rotamer IV match those predicted for conformer **aa1** or **aa2**. Because we have observed quite intense c-type transitions for rotamer IV and only conformer **aa1** is predicted to have a substantial value of *μ*_c_, rotamer IV is identified as conformer **aa1**. On similar grounds, rotamer V was finally identified as the **ag1** conformer. Conformational relative abundances were estimated by measuring transition relative intensities of selected a-, b-, and c-type transitions. For the a-type transitions of conformer **aa1**, which are split into two, the sum of the intensity of each doublet component was used, thus accounting for conformational degeneracy. The resulting conformational relative abundances are **ga1** : **gG1** : **ga2** : **aa1** : **ag1** = 10 : 3 : 2 : 2 : 1, in agreement with predictions from Gibbs free energies at 298 K (see Table 1).

Other conformers of serinol were expected to be observed considering their predicted energies but they were not detected. This can be explained considering that higher-energy conformers can relax to lower-energy ones in the supersonic jet if the barriers between them are low enough (around 1000 cm^–1^ for systems where interconversion can occur *via* several degrees of freedom).^20^ Interconversion barriers have been calculated for higher-energy conformers belonging to the different families of serinol along the corresponding torsional coordinates (see Fig. S2–S5 in the ESI†), and in all cases the barriers have been found to allow relaxation. The low barriers predicted for conformational interconversion reveal a fairly wavy and shallow potential energy surface.

The relative ease of serinol to undertake internal motions is evidenced in the observation of tunneling between two equivalent forms of conformer **aa1**. The determined energy difference between the two tunneling states is very small (∼0.30 MHz), which indicates that the motion mainly involves the hydrogen atoms of the hydroxyl and amino groups. The absence of pure a-type rotational transitions implies that the tunneling pathway proceeds through a structure with zero *μ*_a_, and involves torsions along several coordinates. We have investigated several possibilities, considering clockwise and anticlockwise rotations of the amino group and including pathways going through some of the predicted higher-energy conformers of the **aa** family (Fig. S6 in the ESI†). The lowest energy paths involve anticlockwise rotation of the –NH_2_ group in going from **aa1** to its specular image and passing through a transition state 728 cm^–1^ above **aa1** and then through conformer **aa4**, at 540 cm^–1^ above **aa1**. A similar tunneling motion has been observed in the related molecule glycerol.^21^

The five observed conformations of the polar headgroup of sphingosine are mostly stabilized by a network of N–H···O and O–H···N hydrogen bonds. The most abundant conformer **ga1** exhibits a chain of O–H···N and N–H···O hydrogen bonds, where the amino group simultaneously acts as both a proton donor and an acceptor. Similar networks are displayed by conformers **ga2** and **aa1**. Interestingly, the second most abundant conformer **gG1** is stabilised by three hydrogen bonds O–H···N, N–H···O and O–H···O that form a six-membered cycle involving all functional groups in serinol. The least abundant conformer **ag1** is stabilised by an O–H···N hydrogen bond. The conformational preferences of serinol can be related to hydrogen bond lengths (see Fig. 2), assuming that *ab initio* bond lengths are very good approximations to the actual ones as the difference between experimental and calculated rotational constants is at most 0.9%. In all conformers the O–H···N bond is the shortest, confirming the better ability of the amino group to act as a hydrogen acceptor compared to the hydroxyl group. Similar hydrogen bond networks have been found in the observed conformers of related molecules.^21,22^

**Fig. 2:**
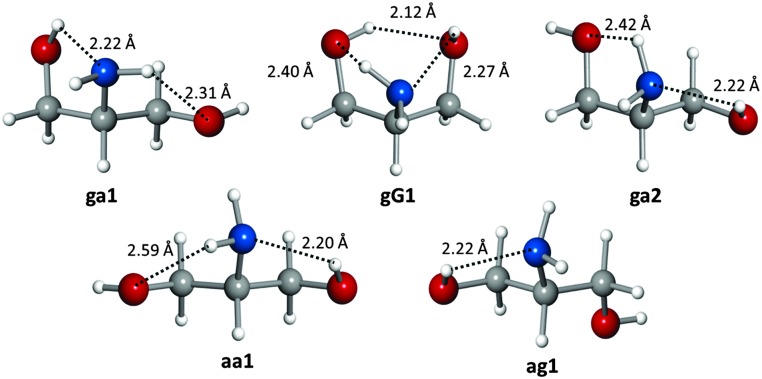
The observed conformers of serinol showing their hydrogen bonds, and the *ab initio* hydrogen bond lengths.

The remarkable conformational variety and relatively flat potential energy surface of the polar headgroup of sphingosine, with many conformers separated by small barriers, seem to indicate that conversion between forms is relatively easy, and perhaps a relevant factor in sphingosine signalling. Many of the calculated barriers are low and could be surmounted under room temperature conditions. Conformer interconversion results in changes in magnitude and direction of the overall dipole moment, which would change sphingosine polarity. For example, conformers **ga1** and **ga2** have predominant dipole moments along the *a* inertial axis, although in opposite directions, while the conformer **gG1** has the dominant dipole moment along the *b* inertial axis. Therefore, depending upon the conformation adopted by the headgroup, sphingosine will display larger polarity in the direction perpendicular to the hydrocarbon tail or parallel to it.

To conclude, five different conformations of serinol have been characterised and their interconversion dynamics have been elucidated by rotational spectroscopy. Assuming that the hydrocarbon tail of sphingosine does not participate in its intramolecular interactions, the study of serinol provides direct and comprehensive information, at the atomic resolution, on the hydrogen bond networks of sphingosine. The interactions and behaviour of other large biomolecules, not amenable to high resolution studies, could thus be inferred from rotational investigations of appropriate smaller biomolecular probes. Evidence of the suitability of this approach could be taken by extrapolation from our observations on amino acids: amino acids with aliphatic hydrocarbon side chains behaved similarly in conformational terms irrespective of the side chain.^23^ Studies of the conformations and interactions of the headgroups of other sphingolipids could be undertaken to help in shedding some light on the different roles of these biomolecules in cellular processes.

This work was supported by the EU FP7 (Marie Curie grant PCIG12-GA-2012-334525), King's College London, Ministerio de Economía y Competitividad (grants CTQ 2013-40717-P and Consolider Ingenio 2010 CSD 2009-00038) and Junta de Castilla y León (Grant VA175U13). The authors acknowledge the use of the computational resources at the Imperial College High Performance Computing facility.
