# Supplementary material for: Intramolecular interactions in the polar headgroup of sphingosine: serinol
Source: Chem Commun (Camb). 2016 Jan 4;52(18):3615–8. doi: 10.1039/c5cc09423b (PMC4766959; doi:10.1039/c5cc09423b)

Electronic Supplementary Material (ESI) for ChemComm.
This journal is © The Royal Society of Chemistry 2015

Supporting Information

Intramolecular Interactions in the Polar Headgroup of Sphingosine: Serinol

D. Loru, I. Peña, J. L. Alonso, M. E. Sanz*

Table S1. Ab initio[a] spectroscopic parameters for the predicted conformers of serinol with energies within 1000 cm^{-1}.

MP2	**ga1**	**ga2**	**ga3**	**ga4**	**ga5**	**gG1**	**gG2**	**gG3**	**gG4**	**aa1**
A^b (MHz)	6084.2	5996.5	6042.6	6033.1	5916.3	4242.0	4094.0	4099.4	4088.4	7738.4
B (MHz)	2278.9	2272.6	2258.1	2235.3	2287.0	3134.0	3237.3	3242.7	3198.2	1977.6
C (MHz)	1997.3	1977.5	1983.2	1951.3	1972.8	2550.2	2479.7	2474.2	2469.0	1699.4
μ_a (D)	-1.67	4.19	1.24	-1.96	0.88	-0.24	2.06	-4.15	-2.37	3.35
μ_b (D)	0.31	-0.83	1.37	0.03	-2.15	-2.62	-0.28	-0.23	1.17	-0.99
μ_c (D)	0.81	-1.37	3.02	0.89	-0.24	-1.51	1.84	1.62	0.55	1.74
μ_{total} (D)	1.88	4.48	3.54	2.15	2.33	3.03	2.77	4.46	2.70	3.90
χ_{aa} (MHz)	-0.34	-4.17	-0.68	-3.99	1.77	-2.60	-2.54	-1.92	-1.57	-3.53
χ_{bb} (MHz)	2.44	2.54	2.47	2.57	-0.24	1.45	0.01	-0.51	-0.84	1.72
χ_{cc} (MHz)	-2.10	1.64	-1.79	1.43	-1.53	1.15	2.53	2.43	2.41	1.80
ΔE_{MP2} (cm^{-1})	0.0	270.8	213.3	472.2	866.5	73.4	525.2	726.7	778.2	368.2
$\Delta E_{MP2+ZPC}$(cm^{-1})	0.0	214.9	247.6	443.3	705.6	145.7	503.3	731.9	825.0	298.3
ΔG^{298} (cm^{-1})	0.0	158.7	276.5	420.5	610.6	114.6	498.7	773.4	911.5	231.5

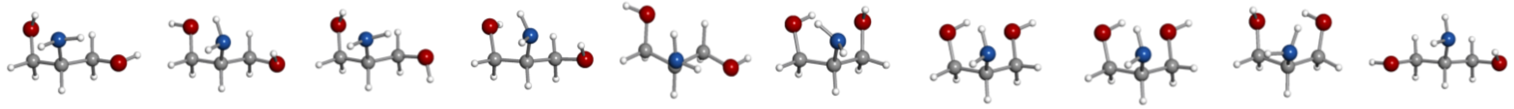

[a] Optimised structures at the MP2/6-311++G(d,p) level, labelled according to the values of the ∠O_1CCC angle (first label) and the ∠CCCO_2 angle (second label) as **G** (+60°), **g** (–60°) and **a** (180°). [b] A, B, C are the rotational constants; χ_{aa}, χ_{bb}, and χ_{cc} are ^{14}N nuclear quadrupole coupling constants; μ_a, μ_b, μ_c are the electric dipole moment components; ΔE_{MP2} are the electronic energies, $\Delta E_{MP2+ZPC}$ are the electronic energies including zero-point corrections, and ΔG^{298} are the Gibbs free energies at 298 K.

Table S1 (cont.). Ab initio[a] spectroscopic parameters for the predicted conformers of serinol with energies within 1000 cm^{-1}.

MP2	**aa2**	**aa3**	**aa4**	**aa5**	**ag1**	**ag2**	**ag3**	**gg1**	**gg2**	**gg3**	**Gg1**	**Gg2**
A^b (MHz)	7642.0	7593.2	7833.3	7874.7	5361.2	5320.1	5438.7	4172.4	4541.3	4101.2	3972.9	3924.7
B (MHz)	1951.8	1957.1	1969.0	1954.9	2387.1	2340.9	2311.3	3166.5	2744.2	3164.6	3283.6	3240.4
C (MHz)	1686.4	1675.5	1702.7	1677.9	1775.3	1759.0	1744.3	2195.6	2069.1	2169.4	1942.5	1942.7
μ_a (D)	-1.95	1.72	0.00	-0.91	3.29	1.64	-1.02	1.87	-0.36	-1.18	-1.32	-1.69
μ_b (D)	-0.43	1.31	-2.01	-0.20	3.22	3.02	2.00	2.18	4.02	1.15	1.89	2.87
μ_c (D)	-0.09	1.74	1.24	-1.14	1.25	-0.56	1.52	0.52	2.21	-0.29	1.57	0.05
μ_{total} (D)	2.00	2.77	2.36	1.47	4.77	3.48	2.71	2.92	4.61	1.68	2.79	3.33
χ_{aa} (MHz)	-3.42	-4.12	1.76	0.52	-4.22	-4.20	-4.21	1.86	0.69	-1.82	1.34	2.78
χ_{bb} (MHz)	1.74	2.05	2.76	2.75	2.32	2.36	2.32	-0.20	1.14	2.17	-2.62	1.97
χ_{cc} (MHz)	1.68	2.07	-4.51	-3.27	1.91	1.84	1.89	-1.66	-1.83	-0.35	1.28	-4.74
ΔE_{MP2} (cm^{-1})	537.5	752.4	912.8	919.1	700.7	717.6	920.7	418.6	913.6	940.4	878.9	965.0
$\Delta E_{MP2+ZPC}$(cm^{-1})	494.5	667.0	718.1	880.1	583.6	674.9	831.8	478.0	883.6	889.7	827.6	935.5
ΔG^{298} (cm^{-1})	457.4	609.3	600.7	831.2	495.1	637.6	741.2	449.7	873.1	825.0	811.6	917.0

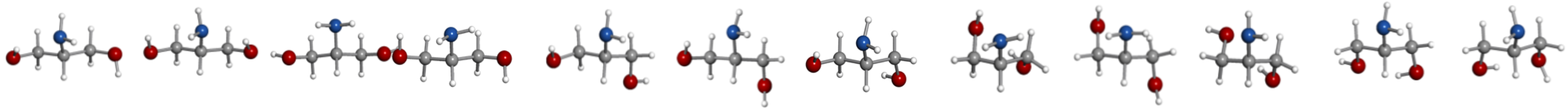

[a] Optimised structures at the MP2/6-311++G(d,p) level, labelled according to the values of the $\angle O_1CCC$ angle (first label) and the $\angle CCCO_2$ angle (second label) as **G** (+60°), **g** (–60°) and **a** (180°). [b] A, B, C are the rotational constants; χ_{aa}, χ_{bb}, and χ_{cc} are ^{14}N nuclear quadrupole coupling constants; μ_a, μ_b, μ_c are the electric dipole moment components; ΔE_{MP2} are the electronic energies, $\Delta E_{MP2+ZPC}$ are the electronic energies including zero-point corrections, and ΔG^{298} are the Gibbs free energies at 298 K.

Table S2. Measured frequencies and residuals (in MHz) for the rotational transitions of conformer **ga1** of serinol.

J'	K'_{-1}	K'_{+1}	J''	K''_{-1}	K''_{+1}	F'	F''	ν_{obs}	$\nu_{obs} - \nu_{cal}$
1	1	1	0	0	0	2	1	8031.002	0.003
						1	1	8031.689	-0.011
						0	1	8029.963	0.016
1	1	0	0	0	0	2	1	8315.049	0.005
						0	1	8314.442	0.009
						1	1	8315.963	0.001
2	0	2	1	0	1	3	2	8477.019	-0.015
2	1	2	1	1	1	3	2	8208.556	0.002
						1	0	8209.239	-0.003
						1	1	8207.487	-0.003
2	1	1	1	1	0	3	2	8776.339	0.036
						2	1	8776.132	-0.031
						1	0	8775.819	0.017
						2	2	8775.547	-0.004
						1	1	8777.329	-0.002
2	2	1	2	1	1	1	1	11353.960	-0.002
2	1	2	1	0	1	3	2	11993.317	-0.010
						2	1	11994.068	-0.005
						1	0	11992.823	-0.006
2	1	1	1	0	1	3	2	12845.114	-0.007
						2	1	12844.439	-0.020
						1	0	12845.427	0.023
2	2	1	2	1	1	3	3	11354.447	0.013
						2	2	11355.283	0.002
						1	1	11353.960	-0.002
3	0	3	2	0	2	4	3	12677.380	-0.002
3	1	3	2	1	2	4	3	12303.497	0.025
						3	2	12303.497	0.025
						3	3	12304.136	0.002
						2	2	12302.581	0.007
3	1	2	2	1	1	4	3	13154.436	-0.007
						3	2	13154.436	-0.022
						3	3	13153.682	-0.022
						2	2	13155.499	-0.004
3	2	2	2	2	1	4	3	12738.552	-0.008
						3	2	12738.552	-0.008
3	2	1	2	2	0	4	3	12799.848	-0.003
						2	1	12799.848	-0.003
3	0	3	2	1	2	4	3	9161.090	0.001
						3	2	9160.468	0.003
						2	1	9161.453	0.011
3	1	3	2	0	2	3	2	15820.464	-0.009
3	1	2	2	0	2	4	3	17522.574	0.007
						3	2	17521.827	0.012
3	2	2	3	1	2	4	4	10938.556	-0.010
						3	3	10939.361	0.009
						2	2	10938.286	-0.004
4	0	4	3	0	3	5	4	16833.653	0.018
4	1	4	3	1	3	5	4	16387.840	0.023
						4	4	16388.516	-0.013
						3	3	16386.954	-0.001
4	1	3	3	1	2	5	4	17520.041	0.005
4	2	3	3	2	2	5	4	16972.767	-0.005
4	2	2	3	2	1	5	4	17123.824	-0.029
4	0	4	3	1	3	5	4	13691.236	-0.023

						4	3	13690.746	0.022
4	2	3	4	1	3	5	5	10391.284	-0.017
						4	4	10392.061	0.001
5	2	4	5	1	4	6	6	9720.480	0.021
						5	5	9720.205	0.009
						4	4	9720.287	-0.023

Table S3. Measured frequencies and residuals (in MHz) for the rotational transitions of conformer **ga2** of serinol.

J'	K'_{-1}	K'_{+1}	J''	K''_{-1}	K''_{+1}	F'	F''	ν_{obs}	$\nu_{obs} - \nu_{cal}$
1	1	1	0	0	0	2	1	7947.389	0.002
						1	1	7948.106	0.002
						0	1	7946.312	-0.002
1	1	0	0	0	0	2	1	8238.674	0.000
						1	1	8239.137	0.003
						0	1	8237.984	-0.001
2	0	2	1	0	1	3	2	8429.507	0.008
						1	0	8428.451	0.023
						1	1	8431.371	0.003
2	1	2	1	1	1	3	2	8154.828	0.004
						2	1	8153.618	0.003
						1	0	8156.171	-0.001
2	1	1	1	1	0	3	2	8737.328	-0.001
						2	1	8736.103	0.000
						1	0	8738.445	-0.001
						2	2	8736.562	0.000
2	1	2	1	0	1	3	2	11879.097	0.006
						2	1	11879.764	-0.011
						1	0	11877.603	0.002
2	2	0	2	1	1	3	3	11189.628	-0.017
						2	2	11191.659	0.000
2	1	1	1	0	1	3	2	12752.881	-0.003
						2	1	12753.288	-0.004
						1	0	12751.542	-0.004
						2	2	12752.112	-0.004
						1	1	12754.487	0.001
2	2	1	2	1	1	3	3	11173.217	-0.005
						2	2	11175.239	-0.010
						1	1	11172.074	-0.021
3	0	3	2	0	2	4	3	12603.427	-0.012
						3	3	12602.166	0.001
						2	2	12605.132	0.001
3	1	3	2	1	2	4	3	12221.987	-0.001
						3	2	12221.647	0.010
						2	1	12221.987	-0.001
						3	3	12221.149	0.005
						2	2	12222.773	0.010
3	1	2	2	1	1	4	3	13095.133	-0.006
						3	2	13094.780	-0.009
						3	3	13094.021	-0.001
						2	2	13096.283	-0.015
3	2	2	2	2	1	4	3	12669.009	-0.004
						3	2	12667.748	-0.006
						2	1	12669.709	-0.005
3	2	1	2	2	0	4	3	12734.358	0.002
						3	2	12733.069	-0.006
						2	1	12735.057	-0.003
3	1	3	2	0	2	4	3	15671.594	0.023
						3	2	15671.992	0.008
						2	1	15671.152	-0.017
3	2	2	3	1	2	4	4	10747.090	-0.005
						3	3	10748.228	0.014
						2	2	10746.704	0.000
3	2	1	3	1	2	4	4	10828.896	0.035
						3	3	10829.950	0.004

						2	2	10828.490	0.008
4	0	4	3	0	3	5	4	16730.459	-0.014
						4	4	16729.205	0.008
						3	3	16732.063	-0.011
4	1	4	3	1	3	5	4	16277.963	-0.032
						4	4	16277.004	-0.010
						3	3	16279.071	-0.009
4	2	3	3	2	2	5	4	16878.980	0.021
						4	3	16878.407	-0.020
4	2	2	3	2	1	5	4	17040.172	0.075
						4	3	17039.540	0.001

Table S4. Measured frequencies and residuals (in MHz) for the rotational transitions of conformer **gG1** of serinol.

J'	K'_{-1}	K'_{+1}	J''	K''_{-1}	K''_{+1}	F'	F''	ν_{obs}	$\nu_{obs} - \nu_{cal}$
1	1	1	0	0	0	2	1	6735.891	0.000
						1	1	6736.263	0.004
						0	1	6735.336	-0.002
1	1	0	0	0	0	2	1	7339.254	0.013
						1	1	7339.597	0.014
						0	1	7338.740	0.011
2	0	2	1	0	1	3	2	11124.882	0.011
						2	1	11124.882	0.011
						1	1	11125.978	0.008
2	1	2	1	1	1	3	2	10712.913	0.013
						2	1	10712.178	0.012
2	1	1	1	1	0	3	2	11919.592	-0.004
						2	1	11918.845	-0.014
						1	0	11920.279	-0.047
2	0	2	1	1	1	3	2	10047.166	0.006
						2	1	10046.090	0.004
						1	0	10048.110	0.006
						2	2	10046.460	0.006
2	1	2	1	0	1	3	2	11790.607	-0.002
						2	1	11790.952	-0.001
						1	0	11789.749	0.001
						2	2	11790.234	-0.009
						1	1	11791.522	-0.000
2	2	1	1	1	0	3	2	15152.992	-0.012
						2	1	15153.421	-0.003
						2	2	15153.761	-0.004
						1	1	15152.227	-0.014
2	2	0	1	1	1	3	2	15947.639	-0.003
						2	1	15947.976	-0.003
						2	2	15948.340	-0.007
						1	1	15946.879	-0.003
2	0	2	1	1	0	3	2	9443.793	-0.016
						2	1	9442.747	-0.015
						1	0	9444.710	-0.002
						2	2	9443.106	0.003
2	1	1	1	0	1	3	2	13600.670	0.015
						2	1	13600.994	0.023
						1	0	13599.826	0.015
						2	2	13600.282	0.022
						1	1	13601.604	0.020
2	2	1	1	1	1	3	2	15756.376	0.020
						2	1	15756.733	-0.015
						2	2	15757.117	0.000
						1	1	15755.562	-0.003
2	2	0	1	1	0	3	2	15344.290	-0.001
						2	1	15344.640	-0.015
						2	2	15344.987	-0.009
						1	1	15343.544	-0.014
3	0	3	2	0	2	4	3	16308.833	0.004
3	0	3	2	1	2	4	3	15643.080	-0.008
						3	2	15642.765	-0.006
						3	3	15642.400	-0.005
						2	2	15643.682	-0.010
3	0	3	2	1	1	4	3	13833.042	0.000
						3	2	13832.737	-0.017

3	1	3	2	0	2	4	3	16629.514	0.031
						3	3	16628.932	0.011
						2	2	16630.403	0.019
3	1	2	2	2	1	4	3	14500.108	-0.005
						3	2	14498.766	-0.007
						2	1	14500.736	-0.002
						3	3	14499.537	0.004

Table S5. Measured frequencies and residuals (in MHz) for the rotational transitions of conformer **aa1** of serinol.

J'	K'_{-1}	K'_{+1}	J''	K''_{-1}	K''_{+1}	F'	F''	v'	v''	ν_{obs}	ν_{obs} - ν_{cal}
1	1	0	0	0	0	2	1	0	0	9648.302	0.000
						2	1	1	1	9648.302	0.000
						1	1	0	0	9648.781	-0.003
						1	1	1	1	9648.781	-0.003
						0	1	0	0	9647.584	0.006
						0	1	1	1	9647.584	0.006
1	1	1	0	0	0	2	1	0	0	9368.394	-0.003
						2	1	1	1	9368.394	-0.003
						1	1	0	0	9368.886	-0.005
						1	1	1	1	9368.886	-0.005
						0	1	0	0	9367.656	-0.009
						0	1	1	1	9367.656	-0.009
2	0	2	1	0	1	3	2	0	1	7305.578	0.001
						3	2	1	0	7306.183	0.017
						1	1	0	1	7307.112	-0.013
						1	1	1	0	7307.705	-0.008
2	1	2	1	1	1	3	2	0	1	7035.831	0.001
						3	2	1	0	7036.426	0.008
						2	1	0	1	7034.821	-0.003
						2	1	1	0	7035.391	-0.022
						1	0	0	1	7036.865	0.013
						1	0	1	0	7037.443	0.002
2	1	1	1	1	0	3	2	0	1	7595.631	-0.010
						3	2	1	0	7596.241	0.011
						2	1	0	1	7594.632	-0.003
						2	1	1	0	7595.203	-0.022
						1	0	0	1	7596.637	-0.016
						1	0	1	0	7597.239	-0.002
2	1	2	1	0	1	3	2	0	0	12746.432	-0.004
						3	2	1	1	12746.432	-0.004
						2	1	0	0	12746.890	-0.005
						2	1	1	1	12746.890	-0.005
						1	0	0	0	12745.261	0.000
						1	0	1	1	12745.261	0.000
						2	2	0	0	12745.924	0.005
						2	2	1	1	12745.924	0.005
						1	1	0	0	12747.688	-0.013
						1	1	1	1	12747.688	-0.013
2	1	1	1	0	1	3	2	0	0	13586.152	0.009
						3	2	1	1	13586.152	0.009
						2	1	0	0	13586.595	0.005
						2	1	1	1	13586.595	0.005
						1	0	0	0	13584.989	0.013
						1	0	1	1	13584.989	0.013
						2	2	0	0	13585.001	0.005
						2	2	1	1	13585.001	0.005
						1	1	0	0	13587.415	0.003
						1	1	1	1	13587.415	0.003
3	0	3	2	0	2	4	3	0	1	10933.392	-0.024
						4	3	1	0	10933.987	-0.018
						3	3	0	1	10932.345	0.004
						3	3	1	0	10932.931	0.002
						2	2	0	1	10934.828	-0.004
						2	2	1	0	10935.424	0.004

3	1	3	2	1	2	4	3	0	1	10547.489	-0.011
						4	3	1	0	10548.091	0.002
						3	2	0	1	10547.214	0.002
						3	2	1	0	10547.792	-0.008
3	1	2	2	1	1	4	3	0	1	11386.982	-0.010
						4	3	1	0	11387.584	0.003
						3	2	0	1	11386.709	0.006
						3	2	1	0	11387.309	0.017
3	2	2	2	2	1	4	3	0	1	10973.630	-0.003
						4	3	1	0	10974.215	0.007
						3	2	0	1	10972.594	0.002
						3	2	1	0	10973.193	0.012
						2	1	0	1	10974.215	0.007
						2	1	1	0	10974.810	0.011
3	2	1	2	2	0	4	3	0	1	11013.706	0.001
						3	2	0	1	11012.667	0.004
						3	2	1	0	11013.266	0.015
3	1	3	2	0	2	4	3	0	0	15988.355	-0.006
						4	3	1	1	15988.355	-0.006
						3	2	0	0	15988.593	0.001
						3	2	1	1	15988.593	0.001
						2	1	0	0	15988.058	-0.002
						2	1	1	1	15988.058	-0.002
4	0	4	3	0	3	5	4	0	1	14531.746	0.014
						5	4	1	0	14532.343	0.023
						4	4	0	1	14530.662	0.027
						4	4	1	0	14531.235	0.012
						3	3	0	1	14533.109	0.019
						3	3	1	0	14533.694	0.015
4	1	4	3	1	3	5	4	0	1	14052.108	-0.024
						5	4	1	0	14052.694	-0.027
						3	3	0	1	14053.190	0.017
						3	3	1	0	14053.794	0.033
4	1	3	3	1	2	5	4	0	1	15170.407	0.002
						5	4	1	0	15170.932	-0.061
5	1	5	4	1	4	6	5	0	1	17547.806	-0.007
						6	5	1	0	17548.386	-0.015

Table S6. Measured frequencies and residuals (in MHz) for the rotational transitions of conformer **ag1** of serinol.

J'	K'_{-1}	K'_{+1}	J''	K''_{-1}	K''_{+1}	F'	F''	ν_{obs}	ν_{obs} - ν_{cal}
1	1	1	0	0	0	2	1	7090.148	-0.001
						1	1	7090.830	0.006
						0	1	7089.126	-0.009
1	1	0	0	0	0	2	1	7694.276	-0.007
						1	1	7694.782	0.007
						0	1	7693.563	0.022
2	0	2	1	0	1	3	2	8176.740	0.020
						1	0	8175.680	0.036
						1	1	8178.567	0.000
2	1	2	1	1	1	3	2	7656.134	-0.008
						2	1	7654.926	-0.012
						1	0	7657.429	-0.020
						1	1	7655.790	0.029
2	1	1	1	1	0	3	2	8864.350	-0.011
						2	1	8863.139	-0.005
						1	0	8865.513	0.009
						2	2	8863.623	-0.015
2	1	2	1	0	1	3	2	10616.074	-0.009
						2	1	10616.719	-0.004
						1	1	10617.548	0.002
2	1	1	1	0	1	3	2	12428.444	0.009
						2	1	12428.905	0.024
						1	0	12427.083	-0.001
						2	2	12427.707	-0.004
3	0	3	2	0	2	4	3	12066.566	-0.010
						3	2	12066.566	-0.010
						3	3	12065.360	0.012
						2	2	12068.210	-0.000
3	1	3	2	1	2	4	3	11434.986	0.001
						2	1	11434.986	0.001
						3	2	11434.647	-0.000
						3	3	11434.134	0.015
						2	2	11435.825	0.012
3	1	2	2	1	1	4	3	13240.013	-0.011
						3	2	13239.668	-0.016
						3	3	13238.936	-0.025
						2	2	13241.118	-0.001
3	2	2	2	2	1	4	3	12390.290	0.010
						3	2	12389.020	-0.007
						2	1	12390.979	0.003
3	2	1	2	2	0	4	3	12713.747	0.007
						3	2	12712.441	0.003
						2	1	12714.455	0.009
3	0	3	2	1	2	4	3	9627.185	-0.017
						3	2	9626.507	-0.007
						3	3	9625.981	-0.004
						2	2	9628.159	0.002
3	1	3	2	0	2	4	3	13874.333	-0.011
						3	2	13874.694	-0.006
						2	1	13873.960	-0.009
						3	3	13873.481	-0.000
						2	2	13875.860	-0.007
4	0	4	3	0	3	5	4	15767.059	0.011
4	1	4	3	1	3	5	4	15164.627	-0.003
4	1	3	3	1	2	5	4	17537.926	-0.033

4	2	3	3	2	2	5	4	16454.991	-0.015
						4	3	16454.411	0.010
						3	2	16454.991	-0.015
						5	4	17207.300	0.019
4	0	4	3	1	3	5	4	13959.270	0.018
						4	3	13958.950	0.013
						3	2	13959.270	0.018

Fig S1. The $2_{1,2} \leftarrow 1_{1,1}$ rotational transition of conformer **aa1** of serinol. Each of the three quadrupole components (labelled with the quantum numbers $F' \leftarrow F''$, $F = I + J$) show small tunneling splittings of about 600 kHz.

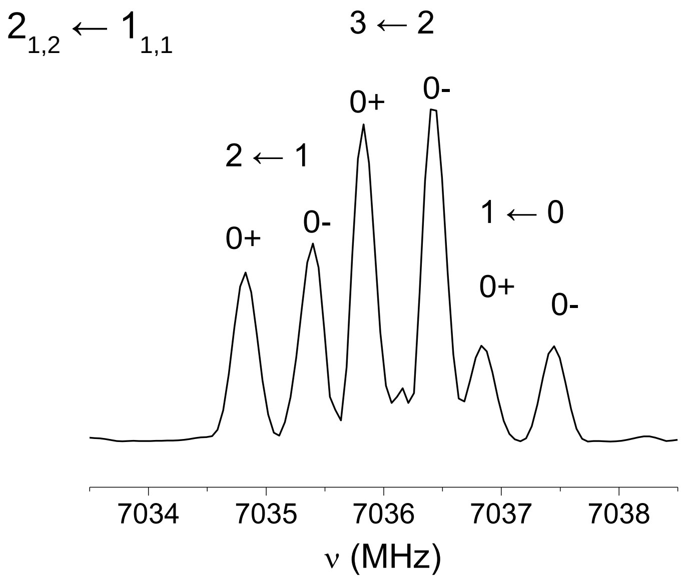

Fig S2. Interconversion barriers between conformers of the **ga** family of serinol calculated at the MP2/6-311++G(d,p) level of theory.

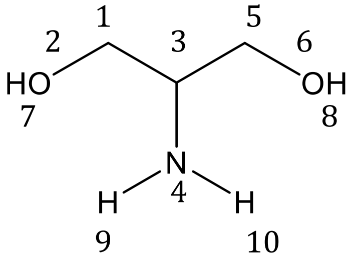

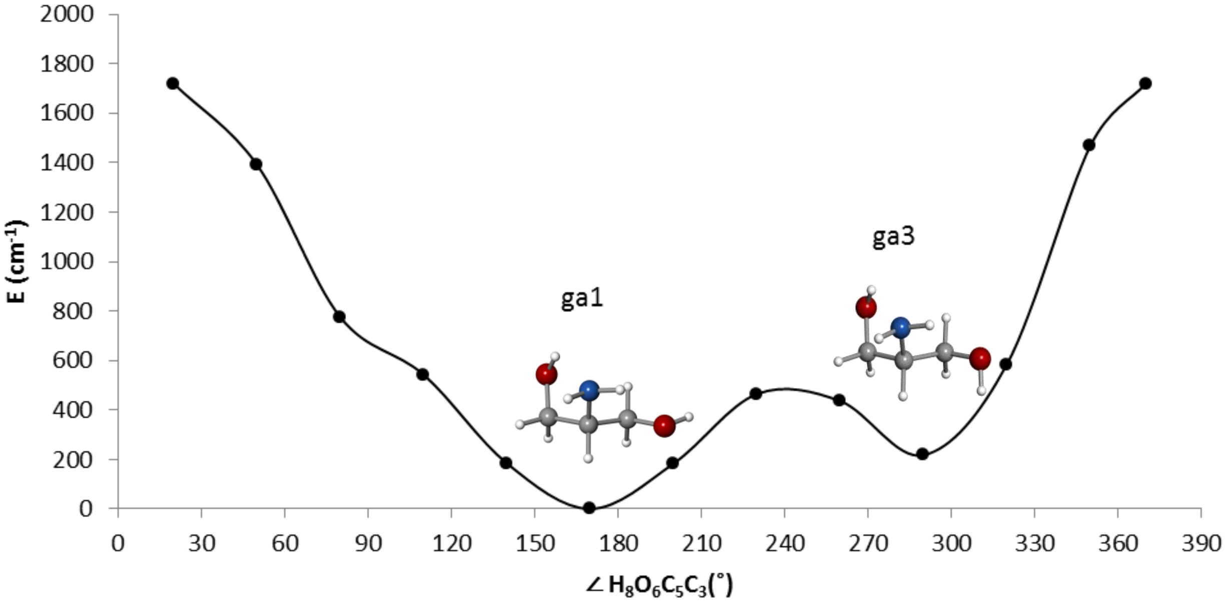

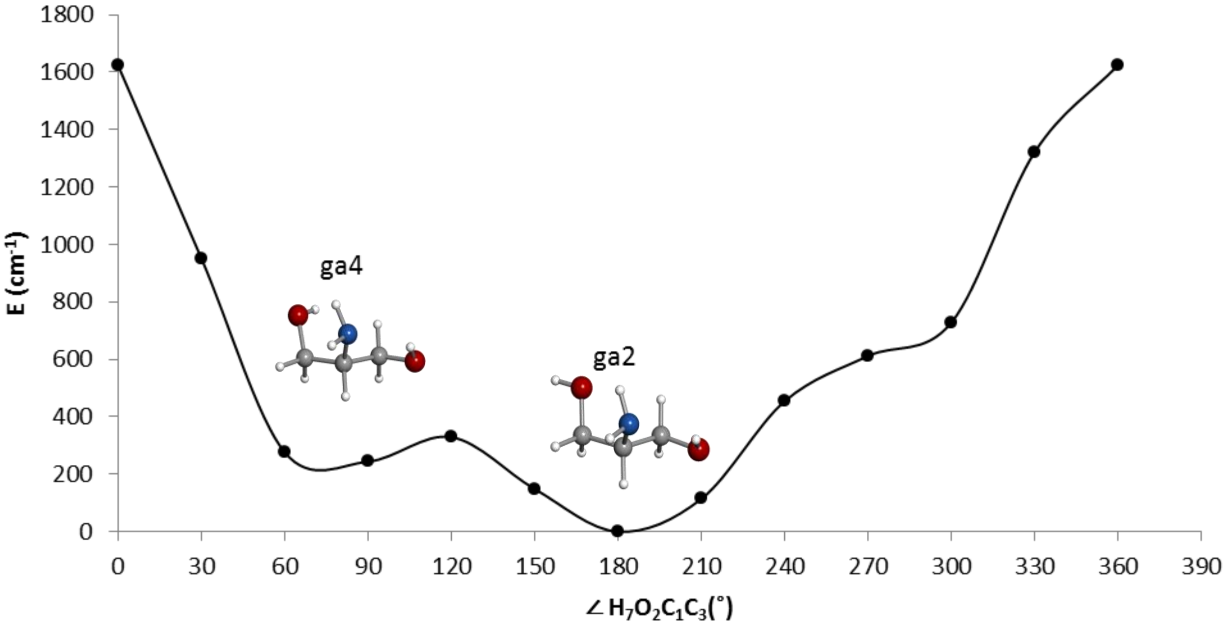

Fig S3. Interconversion barrier between conformers of the **aa** family of serinol calculated at the MP2/6-311++G(d,p) level of theory.

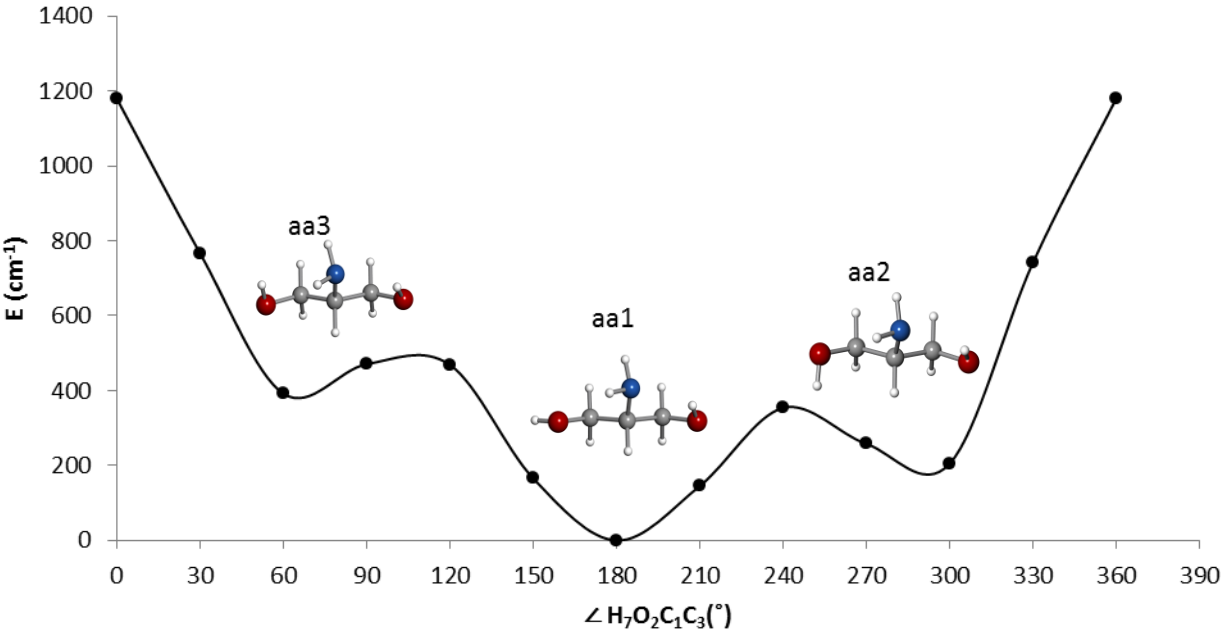

Fig S4. Interconversion barriers between conformers of the **ag** family of serinol calculated at the MP2/6-311++G(d,p) level of theory.

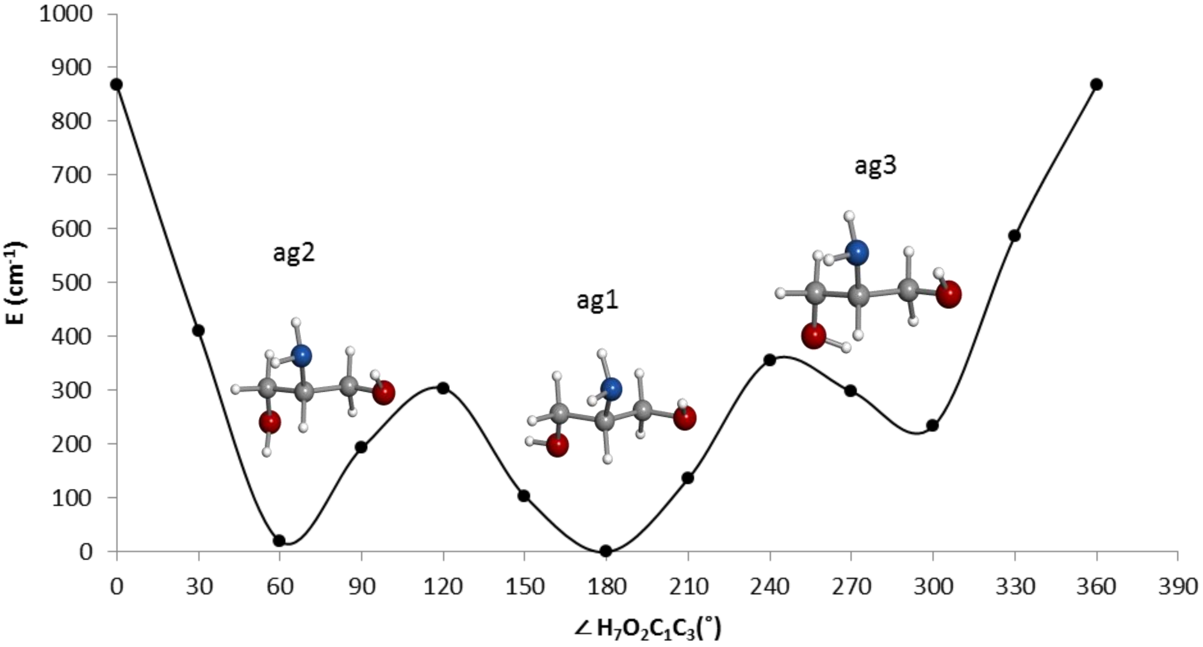

Fig S5. Interconversion barriers between conformers of the **gG** family of serinol calculated at the MP2/6-311++G(d,p) level of theory.

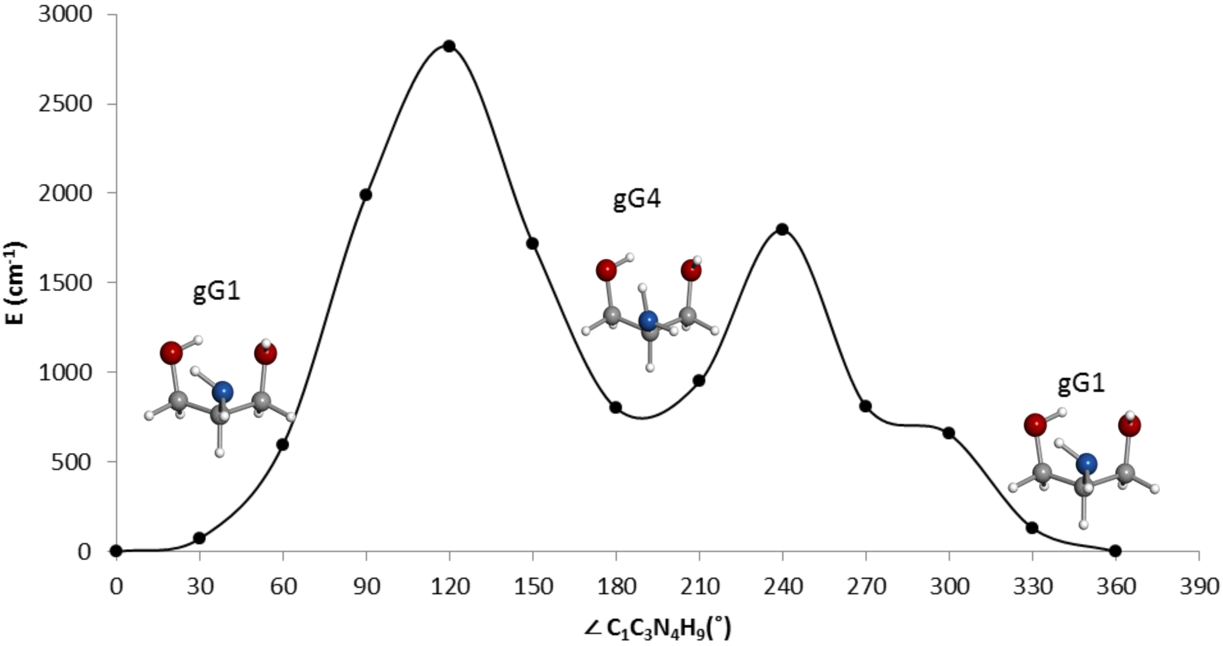

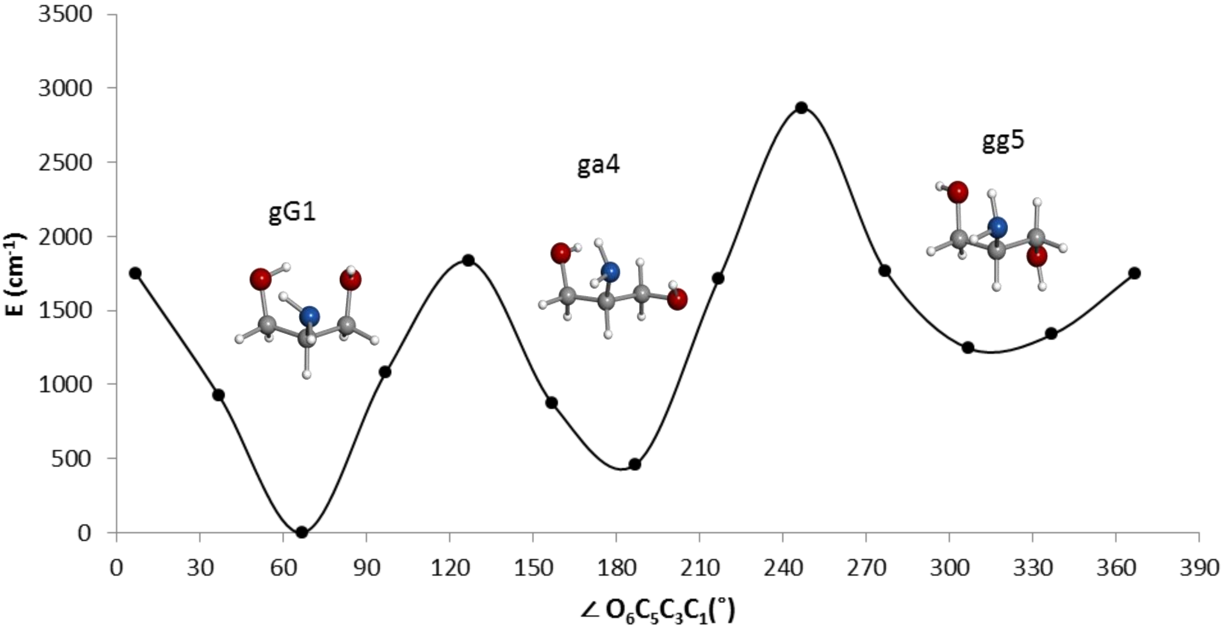

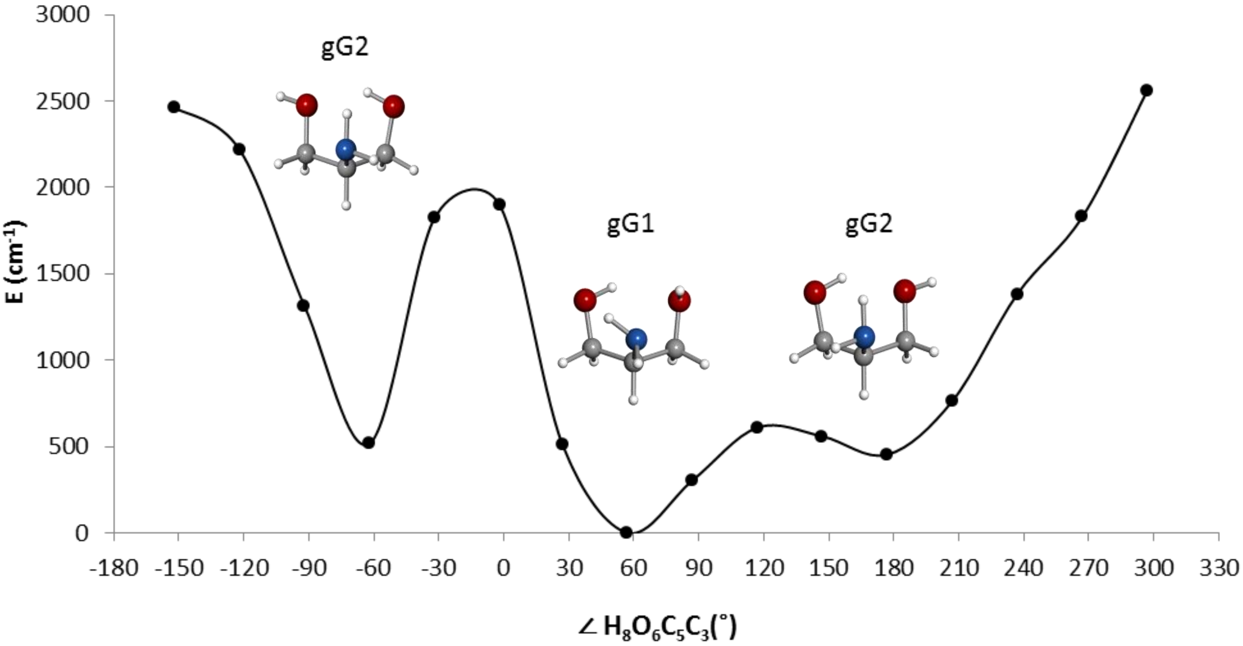

E (cm-1)
∠ H8O6C5C3(°)
gG2
gG1
gG2
3000
2500
2000
1500
1000
500
0
-180 -150 -120 -90 -60 -30 0 30 60 90 120 150 180 210 240 270 300 330

Fig S6. Possible tunneling pathways for the intervconversion of the **aa1** enantiomers of serinol**.** The transition states have been calculated using the Synchronous Transit and Quasi-Newton method (C. Peng, H. B. Schlegel, *Israel J. Chem.*, **33** (1993) 449; C. Peng, P. Y. Ayala, H. B. Schlegel, M. J. Frisch, *J. Comp. Chem.*, **17** (1996) 49) implemented in Gaussian09 at the MP2/6-311++G(d,p) level of theory. Energies are relative to conformer **aa1**. Numbers in parentheses are the zero-point corrected energies.

a)

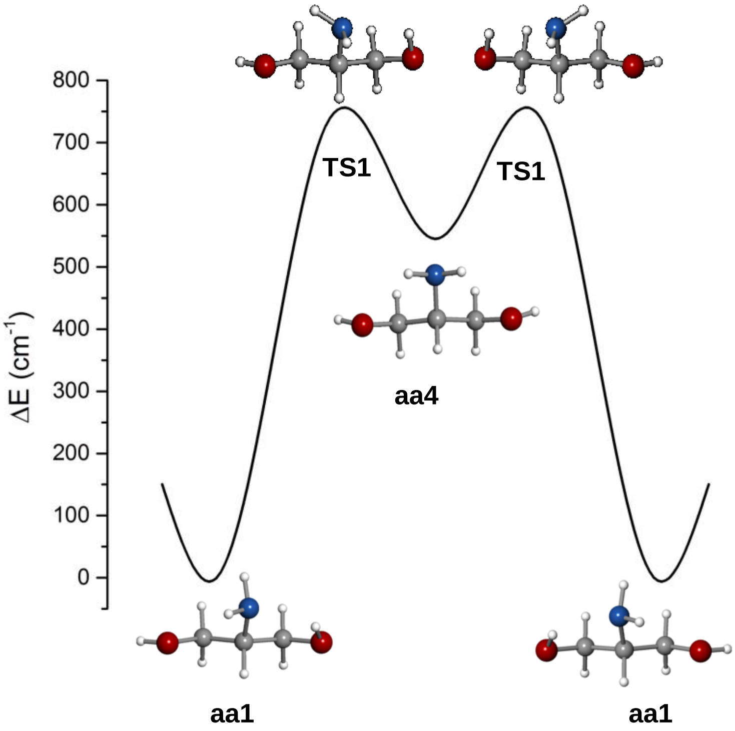

b)

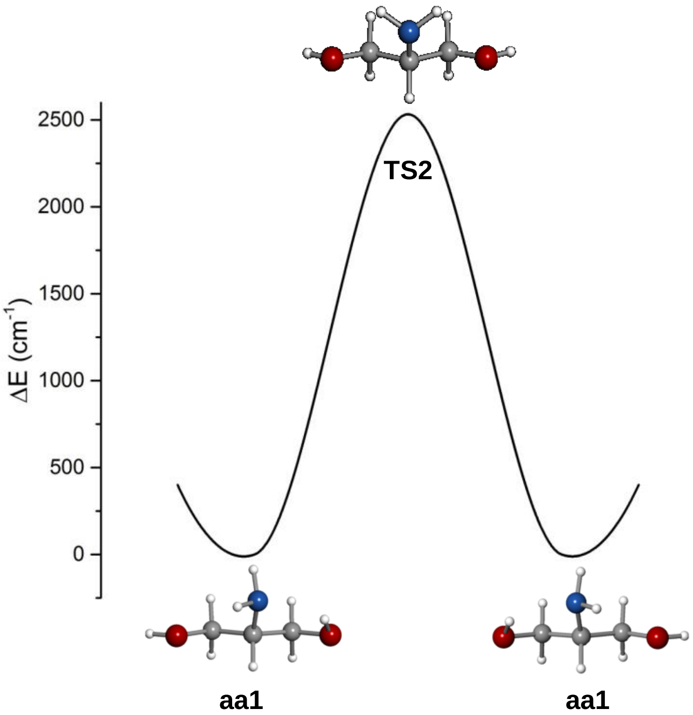

Supplement: Supplementary file 1 [file CC-052-C5CC09423B-s001.pdf]
